# Effect of Cu Content on the Precipitation Behaviors, Mechanical and Corrosion Properties of As-Cast Ti-Cu Alloys

**DOI:** 10.3390/ma15051696

**Published:** 2022-02-24

**Authors:** Zhe Wang, Binguo Fu, Yufeng Wang, Tianshun Dong, Jingkun Li, Guolu Li, Xuebo Zhao, Jinhai Liu, Guixian Zhang

**Affiliations:** 1 School of Materials Science and Engineering, Hebei University of Technology, Tianjin 300401, China; wangz960423@163.com (Z.W.); dongtianshun111@163.com (T.D.); tjjk_zy@126.com (J.L.); liguolu@hebut.edu.cn (G.L.); zhaoxueboliu@126.com (X.Z.); jhliu57@126.com (J.L.); 2 Tianjin Institute of Aerospace Mechanical and Electrical Equipment, Tianjin 300301, China; 3 Key Laboratory of Research and Application of Mould Materials for Glass and Rubber in Hebei Province, Cangzhou 061100, China; andymold@yeah.net

**Keywords:** Ti-Cu alloy, Ti_2_Cu phase, microstructure, mechanical property, corrosion resistance

## Abstract

Ti-Cu alloys have broad application prospects in the biomedical field due to their excellent properties. The properties of Ti-Cu alloys are strongly dependent on Cu content, microstructures, its Ti_2_Cu phase and its preparation process. The aim of this work is to investigate the effect of Cu content on the precipitation behaviors, mechanical and corrosion properties of the as-cast Ti-Cu alloys. The microstructures and phase evolution were characterized by SEM and TEM, and the properties were studied by tensile and electrochemical test. The results show that the volume fraction of Ti_2_Cu phase increases with the increase of Cu content. The Ti_2_Cu phase presents a variety of microscopic morphologies with different Cu content, such as rod, granular, lath and block shaped. The crystal orientation relationships between the Ti_2_Cu and α-Ti matrix in Ti-4Cu and Ti-10Cu alloys are (103)_Ti2Cu/_/(0[1$\bar{1}$11)_α-Ti_, [$\bar{3}$01]_Ti2Cu/_/[2$\bar{1}\bar{1}$0]_α-Ti_, and (103)_Ti2Cu/_/(0002)_α-Ti_, [$\bar{3}$31]_Ti2Cu/_/[1$\bar{2}$10]_α-Ti_, respectively. The tensile strength, Vickers hardness and Young’s modulus of the Ti-Cu alloys increase with the increase of Cu content, whereas the elongation decreases. The fracture morphologies of these alloys reveal ductile, ductile-brittle hybrid, and cleavage brittle mode, respectively. The corrosion resistance of the Ti-Cu alloys in SBF solution can be described as: Ti-4Cu alloy > Ti-10Cu alloy > Ti-7Cu alloy. The volume fraction of Ti_2_Cu phases and the “protective barrier” provided by the fine lath Ti_2_Cu phases strongly affected the electrochemical performances of the alloys.

## 1. Introduction

Titanium alloys are widely used in the biomedical field due to their light weight, high strength, good biocompatibility and corrosion resistance [1,2,3]. However, due to the lack of antibacterial function of titanium, the CP-Ti and TC4 alloys implanted in clinical use are prone to bacterial infection during or after surgeries [4,5]. To date, much attention has been paid to the antibacterial properties of the titanium alloys, and the formation of Ti-Cu alloy by adding Cu element to pure titanium has been proven to be an effective method to improve the antibacterial properties of materials [6,7]. Ti-Cu alloy has good antibacterial properties and can effectively inhibit the formation of bacteria on the surface of the alloy, thereby significantly improving the success rate of joint prosthesis and dental implant surgeries [8]. It is also found that the Ti-Cu alloy can form a good osseointegration with bones, and has a lower bone absorption rate compared with CP-Ti by animal experiments [9]. The excellent antibacterial properties of Ti-Cu alloy can be attributed to the Cu^2+^ released from the Ti_2_Cu phase during the galvanic corrosion process [10]. However, very little information is available on the precipitation mechanism of the Ti_2_Cu phase. Therefore, it is necessary to investigate the precipitation behavior of the alloys.

Cu is an active eutectoid β stabilizer element in titanium alloys, and the melting temperature of the Ti-Cu alloy decreases with the increase of Cu content [11,12]. The addition of Cu element to titanium can favor casting and improve the mechanical properties of the products [13]. In addition to mechanical properties, corrosion resistance is an important consideration for the biomedical materials. Liu et al. found that Ti-Cu alloys had better corrosion resistance than CP-Ti in NaCl solution [14]. However, Osório et al. observed that the increase in Cu content significantly decreased the electrochemical performance of Ti-Cu alloys [15]. Therefore, there are still many controversies concerning the corrosion resistance of the Ti-Cu alloys, and the effect of microstructure, especially the precipitation of the Ti_2_Cu phase on the corrosion performance, needs to be studied in depth.

In this present work, the effects of Cu content (4, 7, 10 wt.%) on the microstructure, mechanical and corrosion properties of as-cast Ti-Cu alloys were systematically investigated. The morphology and formation mechanism of the Ti_2_Cu phase were examined, and the corresponding fracture mechanism and corrosion mechanism were also discussed.

## 2. Materials and Methods

**Figure 1:**
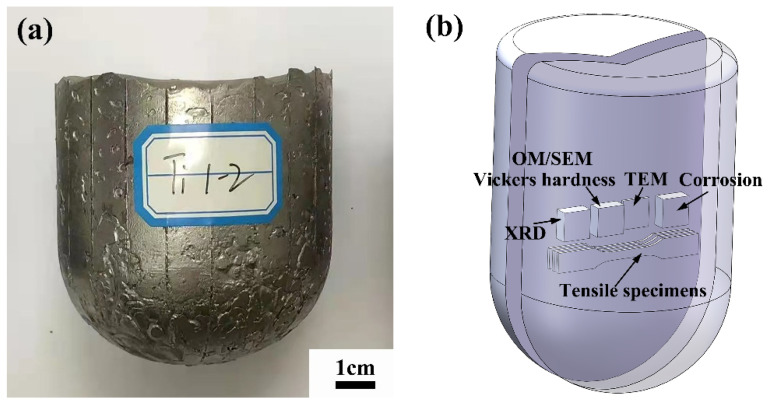
(**a**) Ti-Cu alloy ingot and (**b**) schematic of the sampling locations for microstructure observations, tensile and corrosion testing.

The Ti-xCu alloys with 4, 7, 10 wt.% Cu, respectively, were prepared by a vacuum induction electromagnetic levitation melting furnace with a water-cooled copper crucible. TA1 pure titanium rods (99.95 wt.%) and electrolytic copper (99.99 wt.%) were chosen as the raw materials. The melting stocks weighing 1 kg were melted in an evacuated chamber backfilled with controlled atmosphere of dry high purity argon (99.99%) maintained at 10^3^ Pa. After the raw materials were completely melted, the melt was held for 1 min. Then the power was turned off and the melt was cooled to room temperature in the crucible. Each ingot was flipped and smelted three times to prevent the composition segregation, and the photographs of the ingot are shown in Figure 1a.

The specimens for microstructure observations and performance tests were cut by wire-cut electrical discharge machining (WEDM), and the schematic of the sampling locations is shown in Figure 1b. The phases of the as-cast alloys were characterized by Smartlab (9 kW) X-ray diffractometer (XRD, Rigaku, Akishima, Japan) with monochromatic Cu Kα radiation, and the scanning speed is 6°/min. The microstructure observations and composition analysis were performed by DSX 510 optical microscope (OM, Olympus, Tokyo, Japan), JSM 7610F field emission scanning electron microscope (SEM, JEOL, Tokyo, Japan), Tecnai G2 F30 transmission electron microscope (TEM, FEI, Amsterdam, The Netherland) and X-ray energy dispersive spectroscopy (EDS). Specimens for OM and SEM observations were polished and etched with a mixed solution of 10 mL HF, 50 mL HNO_3_ and 60 mL C_3_H_8_O_3_ for 10 s. Specimens for TEM observations were ground down to 50 μm, punched to Φ 3 mm discs and ion milled. The surface potentials of the as-cast alloys were measured through the atomic force microscope (AFM, Agilent 5500, Santa Clara, CA, USA) in the KPFM mode. Before the AFM experiment, specimens were mechanically polished, and subsequently ultrasonic cleaned with acetone.

**Figure 2:**
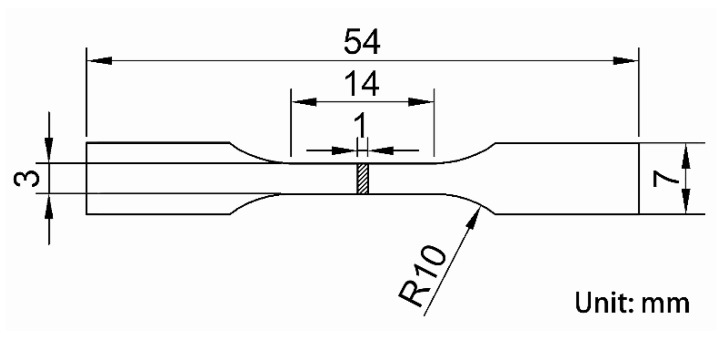
Geometric dimension of the tensile specimen.

Tensile tests were performed at room temperature on an Instron-5848 (Boston, MA, USA) tensile testing machine in air, with a stretching rate of 1 mm/min, according to the ASTM: E8/E8M-16 standard. An extensometer with 10 mm gauge length was used to measure the strain and to exclude the elastic strain of the grips. Young’s modulus was obtained from the slope of the stress-strain curve. The geometric shape of the tensile specimen with 1 mm thickness is shown in Figure 2. Three specimens in each condition were tested, and the statistically averaged values were obtained to analyze the tensile results. The Vickers microhardness was evaluated on an HMV-2000 Vickers hardness tester with a load of 1.918 N and holding time of 20 s. More than ten indentations were performed on each specimen to obtain the mean value.

The electrochemical tests of the specimens were conducted on a beaker containing simulated human body fluid (SBF) solutions at 37 ± 1 °C using the CHI660D three-electrode electrochemical workstation: a platinum electrode as the counter electrode, a saturated calomel electrode (SCE) as the reference electrode, and the testing specimen as the working electrode. The surfaces of the test specimens were polished and then ultrasonically cleaned with acetone. The test geometric area is 1 cm^2^, and the rest is coated with 703 silica gel for insulation. The chemical composition of SBF solution is shown in Table 1. After the components of SBF are fully dissolved, the pH was adjusted 7.4 ± 0.1 using 0.5 mol/L C_4_H_11_NO_3_ (TRIS) and 0.1 mol/L HCl. Before the test starts running, the specimen was placed in the SBF solution and soaked for 24 h to make it passivate naturally. During the potentiodynamic measurements, the electrode was firstly left stabilizing at open circuit potential (OCP) for 1800 s. Afterwards, the electrochemical impedance spectroscopy (EIS) was carried out at an OCP with a 5 mV amplitude sine wave potential and a frequency range of 10^−2^–10^5^ Hz. Finally, the polarization curves were obtained at a scanning range of −0.5–0.15 V (relative to OCP) and the scanning speed was 1 mV/s. Zview software package was used to analyze the impedance data. In order to ensure the accuracy of the experiment, three specimens were measured at each condition.

## 3. Results and Discussion

### 3.1. Microstructure

**Figure 3:**
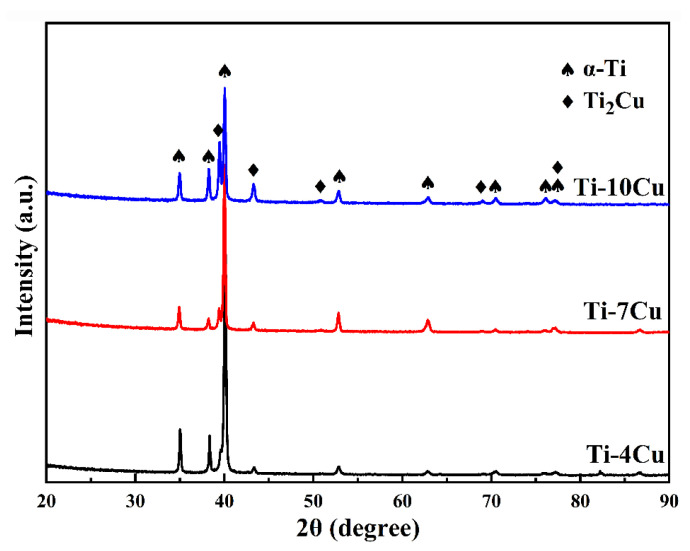
XRD patterns of the as-cast Ti-xCu alloys.

Figure 3 shows the XRD spectra of the as-cast Ti-xCu (x = 4, 7, 10) alloys. It can be seen that the as-cast Ti-Cu alloys are mainly composed of two phases, α-Ti and Ti_2_Cu phase, and no β phase is detected. A previous paper reported that Ti-Cu alloys with low Cu content underwent β → α-Ti + Ti_2_Cu eutectoid transformation, and β phase was difficult to retain at the room temperature, even after quenching [12]. The intensity of the diffraction peaks of the Ti_2_Cu phase gradually increases, indicating that the volume fraction of the Ti_2_Cu phase increases with the increase of Cu content. It can also be observed that the increase of Cu content will promote the α-Ti peaks to shift toward low angle orientations. According to the Bragg’s equation, the deflection of peaks causes the expansion of the α-Ti phase lattice.

**Figure 4:**
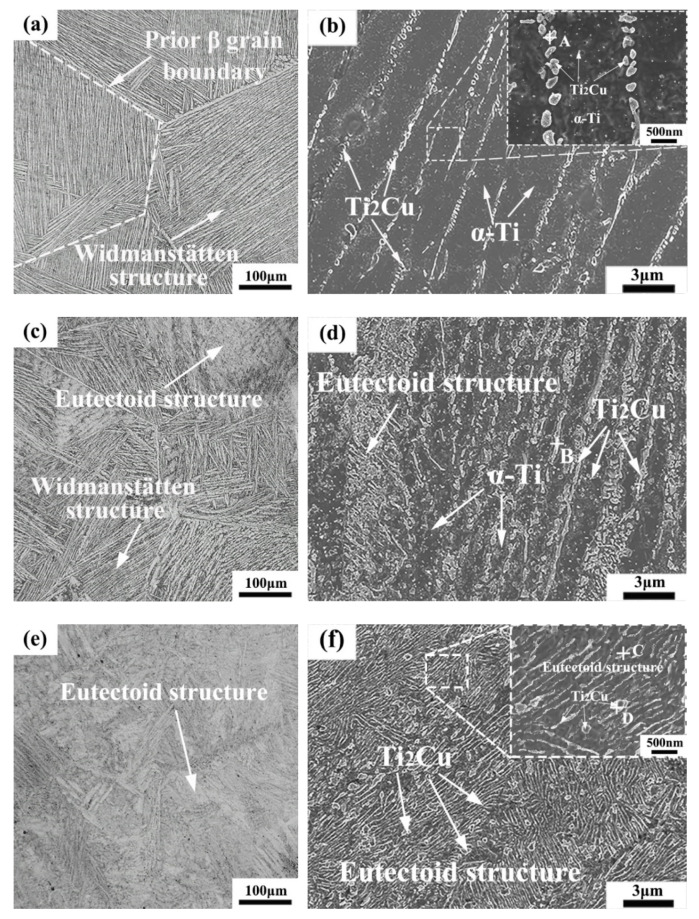
OM and SEM microstructures of the as-cast Ti-xCu alloys: (**a**,**b**) Ti-4Cu alloy; (**c**,**d**) Ti-7Cu alloy; (**e**,**f**) Ti-10Cu alloy.

Figure 4 shows the OM and SEM microstructures of the as-cast Ti-xCu alloys. It can be seen that the OM microstructure of the Ti-4Cu alloy is a Widmanstätten structure, which consists of equiaxed prior β grains and lamellar α-Ti phase (Figure 4a). With the increase of Cu content, the OM microstructure of the Ti-7Cu alloy changes to a mixed microstructure of eutectoid structure and Widmanstätten structure with typical basket weave features (Figure 4c), and the Ti-10Cu alloy is typical eutectoid structure (Figure 4e). Figure 4b shows the SEM microstructure of the Ti-4Cu alloy. It can be seen that lamellar α-Ti and some rod-shaped phases are precipitated at the grain boundaries of the lamellar structures. However, some fine granular particles were formed in the internal grains. EDS of the precipitate and the analysis result is presented in Table 2. According to the EDS and XRD (Figure 3) analysis, the rod-shaped and granular precipitates can be confirmed to be the Ti_2_Cu phase. Similarly, the Ti_2_Cu phases in Ti-7Cu and Ti-10Cu alloys can also be identified. However, the morphologies of the Ti_2_Cu phase are different with the differences of Cu content. In the Ti-7Cu alloy, the Ti_2_Cu phase exhibits both lath and rod morphologies, and some fine granular precipitates were also found inside the α-Ti phase grains (Figure 4d), while it exhibits both lath and block morphologies in the Ti-10Cu alloy (Figure 4f). From the above SEM microstructures, it can also be inferred that the Ti_2_Cu phases increases with the increase of Cu content.

**Figure 5:**
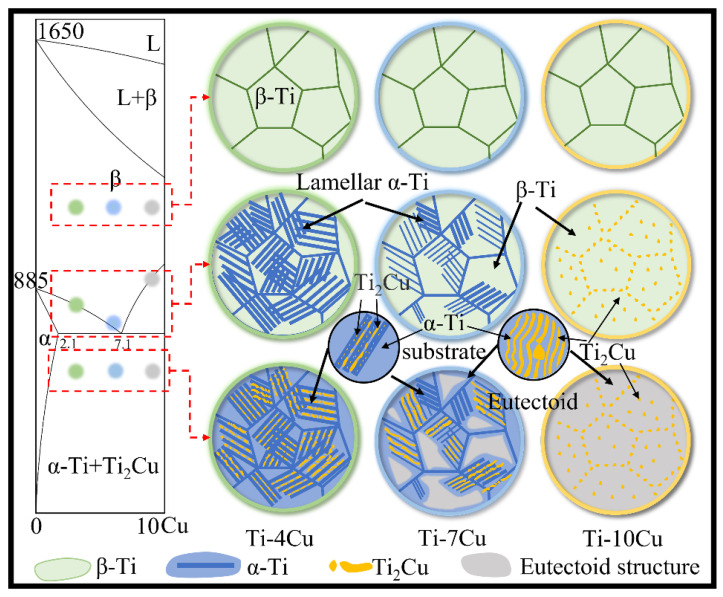
Section of Ti-Cu phase diagram and schematic views of microstructure transformation of Ti-xCu alloys during solidification.

Figure 5 shows the section of Ti-Cu phase diagram and schematic views of the microstructure transformation of Ti-xCu alloys during the solidification process. According to the Ti-Cu binary alloy equilibrium phase diagram [17], the Ti-4Cu, Ti-7Cu and Ti-10Cu alloys correspond to the hypo-eutectoid, near eutectoid and hyper-eutectoid regions, respectively. Solidification occurs by the formation of primary β-Ti grains, and the Ti-Cu alloys are composed of β-Ti single-phase region at the high temperature. Then, the microstructure transformations of Ti-Cu alloys are different with the differences of Cu content, and the schematic views of the microstructures are shown in Figure 5. For the Ti-4Cu alloy, the lamellar pro-eutectoid α-Ti phases precipitate along the prior β-Ti grain boundaries and grow into the grains to form the Widmanstätten structure. As the temperature drops to 790 °C, the residual β-Ti phase undergoes eutectoid transformation into the α-Ti and rod-shaped Ti_2_Cu phase. Due to the difference in the precipitation sequences, more Cu elements are dissolved in the pro-eutectoid α-Ti phases than in the eutectoid α-Ti phases. When the temperature continues to decrease, the pro-eutectoid α-Ti phase will precipitate finely dispersed granular phases due to the oversaturated Cu. As a near-eutectoid alloy, the amount of pro-eutectoid α phases and the temperature interval of the (β+α) two-phase region of the Ti-7Cu alloy are smaller than those of the Ti-4Cu alloy. Therefore, Ti-7Cu alloy contains the Widmanstätten and eutectoid structure, and the Ti_2_Cu phases formed by the eutectoid reaction exhibit both lath and rod morphologies. Due to the presence of more supersaturated Cu in the eutectoid α-Ti phases of the Ti-7Cu alloy, more dispersed fine granular phases will be precipitated in the eutectoid α-Ti phase during the subsequent cooling to room temperature. The Ti-10Cu alloy is a hyper-eutectoid alloy, and it can be seen that the pro-eutectoid block Ti_2_Cu phases are precipitated at the prior β grain boundaries, or internal grains. As the temperature decreases, the β phase undergoes eutectoid transformation, and the α-Ti and lath Ti_2_Cu phases in the eutectoid structure present a layered distribution.

### 3.2. Ti_2_Cu Precipitation

**Figure 6:**
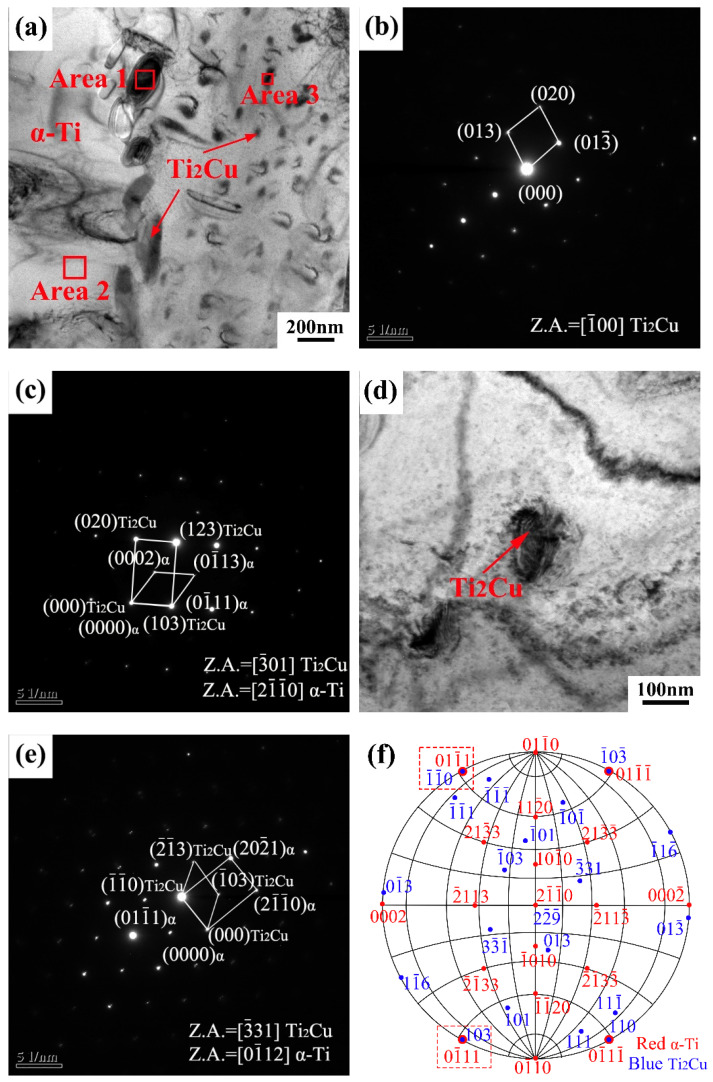
(**a**) Bright field TEM image of the as-cast Ti-4Cu alloy; (**b**) SAED pattern taken from the area 1 in (**a**); (**c**) the complex SAED pattern of the rod-shaped Ti_2_Cu and α phase; (**d**) bright field TEM image of the granular Ti_2_Cu phase; (**e**) the complex SAED pattern of the granular Ti_2_Cu and α phase taken from in (**d**); (**f**) stereogram showing the parallel planes of Ti_2_Cu and α.

The TEM images of the as-cast Ti-4Cu alloy are shown in Figure 6. It can be seen that the rod-shaped and granular precipitates are distributed in the α-Ti matrix (Figure 6a). Figure 6b shows the selected area electron diffraction (SAED) pattern of the rod-shaped phase in Figure 6a, and the corresponding EDS quantitative analysis result (Area 1) is presented in Table 3. The analysis indicates that the rod-shaped precipitate is Ti_2_Cu phase, which presents a tetragonal structure (a = 0.29438 nm, c = 1.0786 nm) [18,19]. Figure 6c presents the complex SAED pattern of the rod-shaped Ti_2_Cu and α-Ti phase, and the orientation relationship between the Ti_2_Cu phase and α-Ti matrix can be described as (103)_Ti2Cu/_/(0$\bar{1}$11)_α-Ti_, [$\bar{3}$01]_Ti2Cu/_/[2$\bar{1}$$\bar{1}$0]_α-Ti_. The same orientation relationship has been reported in the literature [19] by Sun et al. The magnified bright field TEM image of the granular phase is shown in Figure 6d. It can be seen that the size of the particle is about 100 nm. Combined with the EDS analysis result (Area 3) presented in Table 3, the granular precipitate can also be identified as Ti_2_Cu phase. Figure 6e shows the complex SAED pattern of the granular Ti_2_Cu and α phase, and a new orientation relationship between the Ti_2_Cu phase and α-Ti matrix can be described as ($\bar{1}$$\bar{1}$0)_Ti2Cu/_/(01$\bar{1}$1)_α-Ti_, [$\bar{3}$31]_Ti2Cu/_/[0$\bar{1}$12]_α-Ti_. The orientation relationship between the Ti_2_Cu phase and the α-Ti phase was further analyzed using the polar stereographic projection technique [20], as shown in Figure 6f. It can be seen that (103)_Ti2Cu/_/(0$\bar{1}$11)_α-Ti_ orientation relationship is fixed on the outer circumference, and another orientation relationship (($\bar{1}$$\bar{1}$0)_Ti2Cu/_/(01$\bar{1}$1)_α-Ti_) also appears on the outer circumference. According to the rotational symmetry of the crystal, (103)_Ti2Cu/_/(0$\bar{1}$11)_α-Ti_, [$\bar{3}$01]_Ti2Cu/_/[2$\bar{1}$$\bar{1}$0]_α-Ti_ and ($\bar{1}$$\bar{1}$0)_Ti2Cu/_/(01$\bar{1}$1)_α-Ti_, [$\bar{3}$31]_Ti2Cu/_/[0$\bar{1}$12]_α-Ti_ orientation relationships are equivalent.

**Figure 7:**
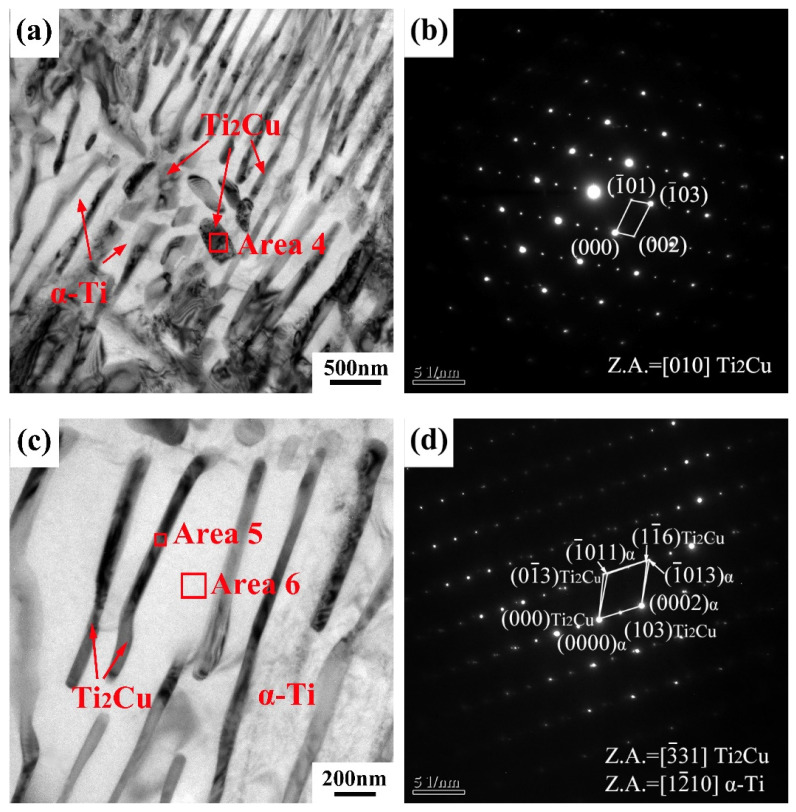
(**a**,**c**) bright field TEM images of as-cast Ti-10Cu alloy; (**b**) SAED pattern taken from the area 4 in (**a**); (**d**) the complex SAED pattern of the lath Ti_2_Cu (Area 5) and α phase taken from in (**c**).

Figure 7 shows the TEM images of the as-cast Ti-10Cu alloy. The microstructure of the Ti-10Cu alloy is composed of pro-eutectoid block and lath precipitates, as shown in Figure 7a. The pro-eutectoid block precipitate can be identified as Ti_2_Cu phase according to the SAED pattern (Figure 7b) and EDS (Table 3) analysis. Figure 7c shows the eutectoid microstructure of the Ti-10Cu alloy. It can be seen that the black lath precipitate is the Ti_2_Cu phase, and the matrix is the α-Ti phase, according to the EDS analysis. The complex SAED pattern of the lath Ti_2_Cu phase and α phase is shown in Figure 7d, and the orientation relationship between the Ti_2_Cu phase and α-Ti matrix is established as (103)_Ti2Cu/_/(0002)_α-Ti_, [$\bar{3}$31]_Ti2Cu/_/[1$\bar{2}$10]_α-Ti_. Williams et al. also found this crystal orientation relationship in Ti-Cu alloys [21].

### 3.3. Mechanical Properties

**Figure 8:**
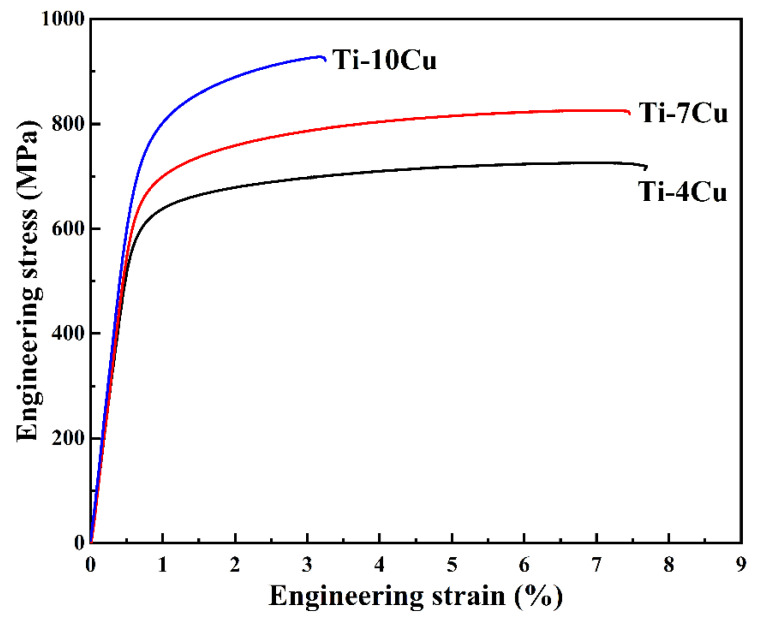
Tensile stress-strain curves of the as-cast Ti-xCu alloys.

Figure 8 shows the tensile engineering stress-strain curves of the as-cast Ti-xCu alloys, and the values of the mechanical properties are listed in Table 4. It can be seen that the ultimate tensile strength (UTS) and yield strength (YS) of the alloys increase with the increase of Cu content, whereas the elongation decreases. The UTS and YS of the Ti-10Cu alloy are 902.7 MPa and 749.5 MPa, which are 25.6% and 22.4% higher than that of Ti-4Cu alloy, respectively. However, the elongation with 2.6%, is 61.2% lower than that of Ti-4Cu alloy. The Vickers hardness and Young’s modulus of the alloys also increase with the increase of Cu content, as seen in Table 4. The strengthening effect of Cu addition to titanium alloys has been reported in many literatures, and solid solution strengthening and precipitation of intermetallic compounds strengthening mechanisms were proposed [7]. With the increase of Cu content in Ti-Cu alloys, both the solid solution Cu element in α-Ti matrix and the precipitated Ti_2_Cu phase increase. As a result, the UTS, YS, and microhardness are increased, and the elongation is decreased. It is known that the Young’s modulus of the alloys is dependent on the alloy phase, and intermetallic compounds generally have higher elastic modulus than the constituting elements. Therefore, the Young’s modulus of Ti-Cu alloys also increases with the increase of Cu content. This result is in good agreement with that reported by Kikuchi et al. [22], which showed that Cu increased the Young’s modulus of titanium by 20% when its content was 30 wt.%.

**Figure 9:**
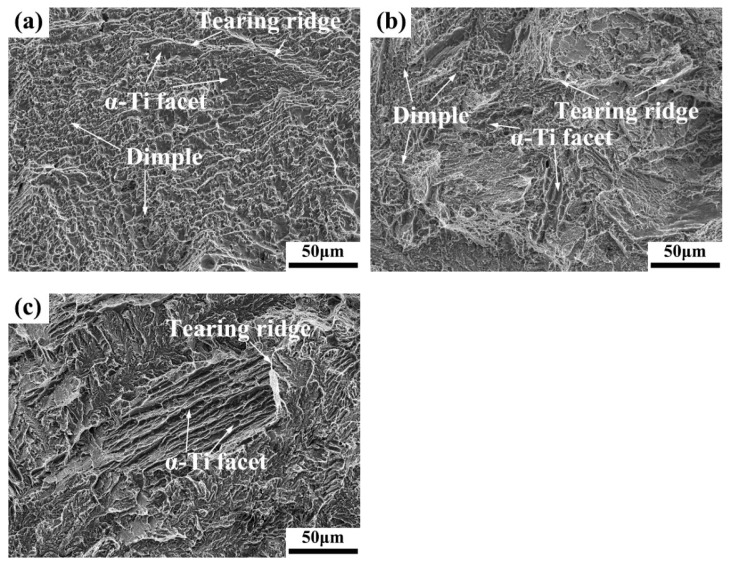
SEM fractographs of the tensile test specimens: (**a**) Ti-4Cu alloy; (**b**) Ti-7Cu alloy; (**c**) Ti-10Cu alloy.

Figure 9 shows the tensile fracture features of the as-cast Ti-Cu alloys at room temperature. It can be seen that the fracture surfaces of the Ti-4Cu alloy are a mixed morphology formed by lots of dimples with different sizes, finer flat cleavage facets, as well as numerous tearing ridges, corresponding to good plasticity of the as-cast Ti-4Cu alloy (Figure 9a). The fracture features of the Ti-7Cu alloy are similar to those of Ti-4Cu alloy, as seen in Figure 9b. However, the smaller number of dimples and the larger number and size of cleavage facets confirm that it is a ductile-brittle hybrid fracture mechanism. The fracture morphology of the Ti-10Cu alloy is entirely composed of cleavage surfaces caused by the brittle behavior of the Ti_2_Cu intermetallic phases, and few tearing ridges appear, as shown in Figure 9c. The fracture mechanism of the Ti-10Cu alloy can be determined as a cleavage fracture mode, showing the lowest plasticity.

### 3.4. Electrochemical Properties

**Figure 10:**
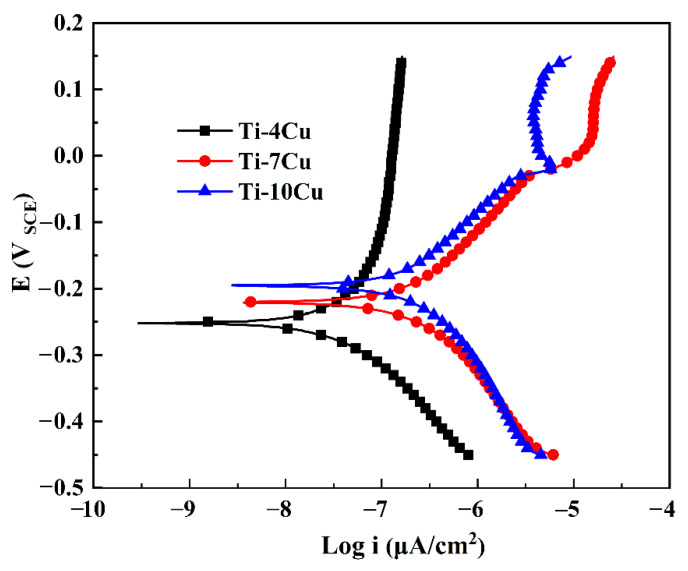
Polarization curves of the as-cast Ti-xCu alloys in SBF solution.

In order to analyze the effect of Cu content on the corrosion properties of the as-cast Ti-xCu alloys, the electrochemical tests of the specimens were carried out in SBF solutions. Figure 10 shows the polarization curves of the alloys. It can be seen that all the alloys show significant activation-passivation characteristics. In the cathodic polarization zone, below the self-corrosion potential, the current density on the surface of the alloy decreases with the increase of the applied voltage, and oxygen absorption corrosion occurs. In the active dissolution zone, when the corrosion current density reaches the minimum value, the corresponding potential is the self-corrosion potential (E_corr_) [23]. As the corrosion potential continues to increase, the corrosion current increases substantially linearly, and the alloys undergo rapid active dissolution within this interval. The cathodic polarization zone and the active dissolution zone basically conform to the characteristics of the Tafel curve. The polarization curves show a linear relationship in the “Tafel area”, and the slope, which is the corrosion current density (i_corr_), can be obtained by fitting the Tafel curve [23]. After the anodic polarization curve has experienced a small active dissolution zone, the corrosion potential reaches a certain value, and the alloy quickly enters the passivation zone. With the continuous increase of the potential, the corrosion current appears to be basically unchanged or increase slightly and slowly. During this process, the passive film on the surface of the alloy tends to be in a stable state, and the corrosion rate is extremely small. Compared with Ti-4Cu alloy, Ti-7Cu and Ti-10Cu alloys have an over passivation zone. In the over passivation zone, as the applied potential continues to increase, the current density begins to increase rapidly, the surface of the alloy begins to enter the active dissolution state from the passivation state, and the corrosion rate of the alloy increases rapidly again.

The values of the i_corr_ and E_corr_ of the Ti-Cu alloys calculated from the polarization curves (Figure 10) are shown in Table 5. The i_corr_ represents the corrosion rate of the material during the corrosion process. A smaller value of i_corr_ indicates that the alloy is more resistant to corrosion. The E_corr_ reflects the thermodynamic tendency of the electrode. The more positive the value of E_corr_, the smaller the tendency for corrosion to occur. The E_corr_ values of the Ti-Cu alloys with different Cu content are relatively close, while the i_corr_ value of Ti-4Cu alloy (0.043 μA/cm^2^) is smaller than that of Ti-10Cu alloy (0.276 μA/cm^2^) and Ti-7Cu alloy (0.413 μA/cm^2^), as shown in Table 5. Therefore, the corrosion resistance of the Ti-Cu alloys in SBF solution can be described as: Ti-4Cu alloy > Ti-10Cu alloy > Ti-7Cu alloy. Compared with the results of the electrochemical property of commercially pure titanium in the SBF solution reported by Yetim, the values of E_corr_ and i_corr_ of CP titanium are −0.491 V and 2.0 μA/cm^2^, respectively, which showed the Ti-Cu alloys had better corrosion resistance than CP-Ti in the SBF solution [24].

**Figure 11:**
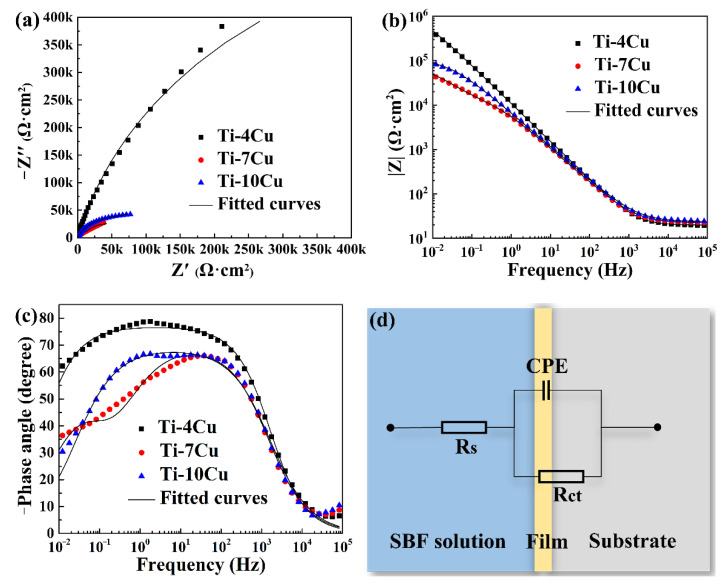
Electrochemical impedance spectrum (EIS) plots of the as-cast Ti-xCu alloys: (**a**) Nyquist plots, (**b**) Bode plots, (**c**) Bode phase angle plots, (**d**) equivalent circuits used for fitting the EIS.

The EIS curves of the as-cast Ti-Cu alloys are shown in Figure 11. Figure 11a shows the Nyquist diagram. It can be seen that the curves of the as-cast Ti-Cu alloys with different Cu content are all circular or arc-shaped. The semicircle represents the capacitive reactance arc created by the parallel circuit of the capacitor and the charge transfer resistor. The diameter of the curve represents the corrosion resistance of the Ti-Cu alloys. The larger the diameter of the curve, the better the corrosion resistance of the alloy [25]. The diameter of the Ti-4Cu alloy test curve is larger than that of the Ti-10Cu alloy curve, indicating that the corrosion resistance of Ti-4Cu alloy is stronger than that of Ti-10Cu alloy. Similarly, the corrosion resistance of Ti-10Cu alloy is stronger than that of Ti-7Cu alloy. Figure 11b shows the Bode plots of the as-cast Ti-Cu alloys. The Bode plot is the curve of the impedance modulus (|Z|) as a function of the disturbance frequency. Generally, the larger the value of |Z| in the low frequency region (<10 Hz), the better the corrosion resistance [26]. The Ti-4Cu alloy has the largest |Z| value at 10 mHz in the low frequency region, showing the strongest corrosion resistance. This is consistent with the previous analysis. Figure 11c shows the Bode phase angle plots of the as-cast alloys. The Bode phase angle plot is the curve of the phase angle as a function of the disturbance frequency. It can be seen that the phase angles of the alloys with different Cu content have no negative values in the low frequency region, indicating that the surfaces of the three alloys can form stable passivation films. Ti-4Cu alloy has the best corrosion resistance due to the largest phase angle peak and peak width, whereas Ti-7Cu alloy has the worst corrosion resistance. Once again, the corrosion resistance of Ti-4Cu alloy > Ti-10Cu alloy > Ti-7Cu is proved. In order to quantitatively characterize the corrosion resistance of the specimens, the equivalent circuit is established by fitting the EIS curves with Zview software, as shown in Figure 11d. The corresponding electrochemical parameters are shown in Table 6. It can be seen that the solution resistance (R_s_) values are at the same order, while the values of the charge transfer resistance (R_ct_) are significantly different. The larger the R_ct_ value, the better the corrosion resistance [27]. The R_ct_ value of Ti-4Cu alloy is 0.537 MΩ·cm^2^, which is ten times that of Ti-7Cu alloy and five times that of Ti-10Cu alloy, indicating that its corrosion resistance is the best. Compared with the R_ct_ value of the CP-Ti presented in Ref. [28], the Ti-Cu alloys also showed better corrosion resistance than the CP-Ti in the SBF solution.

**Figure 12:**
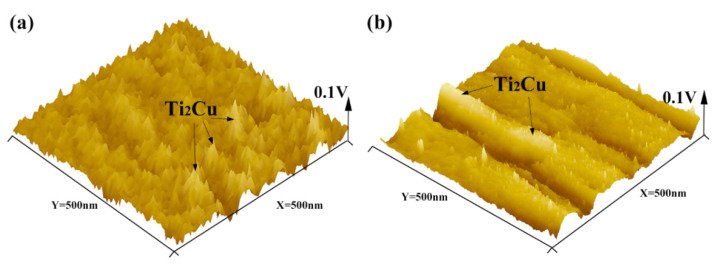
The 3D AFM images and surface potentials of the as-cast Ti-xCu alloys: (**a**) Ti-4Cu alloy, (**b**) Ti-10Cu alloy.

In order to clarify the corrosion mechanism, the surface volt potential maps of the as-cast Ti-4Cu and Ti-10Cu alloys are acquired by AFM in the KPFM mode, as indicated in Figure 12. It can be seen that the Ti_2_Cu phases show more active potential than the α-Ti matrix. Undoubtedly, a number of micro-galvanic corrosion are formed and the α-Ti matrix would act as micro-anode to erode preferentially. In Ti-10Cu and Ti-7Cu alloys, more Ti_2_Cu phases were formed than in the Ti-4Cu alloy (Figure 4), which considerably increased the galvanic corrosion effect and decreased the general corrosion resistance (Figure 11). Similar results were also obtained by Pina et al. [29] when studying the electrochemical and tribo-electrochemical characterization of titanium-copper biomedical alloys. Although it contains more Ti_2_Cu phase in Ti-10Cu alloy than in Ti-7Cu alloy, its corrosion performance is better than that of Ti-7Cu alloy. This can be attributed to the uniform distribution and smaller inter phase spacing of the lath Ti_2_Cu phase. It would provide an “protective barrier” modifying the cathode/anode area ratio between the Ti_2_Cu and α-Ti phases which could minimize the galvanic effects and improve the corrosion resistance [30,31].

## 4. Conclusions

(1) The as-cast Ti-Cu alloys are mainly composed of α-Ti and Ti_2_Cu phase. The volume fraction of Ti_2_Cu phase increases with the increase of Cu content;

(2) The microstructures of the as-cast alloys are Widmanstätten structure, eutectoid and Widmanstätten mixed structure, and eutectoid structure, respectively. The morphologies of the Ti_2_Cu phases change from (rod + granular), (lath + rod + granular) to (lath + block) shaped, with the increase of Cu content;

(3) The orientation relationship between the Ti_2_Cu phase and α-Ti matrix of the Ti-4Cu alloy can be identified as (103)_Ti2Cu/_/(0$\bar{1}$11)_α-Ti_, [$\bar{3}$01]_Ti2Cu/_/[2$\bar{1}$$\bar{1}$0]_α-Ti_. However, in the Ti-10Cu alloy, the orientation relationship between the two phases is established as (103)_Ti2Cu/_/(0002)_α-Ti_, [$\bar{3}$31]_Ti2Cu/_/[1$\bar{2}$10]_α-Ti_;

(4) The UTS, YS, Vickers hardness and Young’s modulus of the Ti-Cu alloys increase with the increase of Cu content, whereas the elongation decreases. The fractographic analyses indicated that the fracture mode changed from ductile, ductile-brittle hybrid to cleavage brittle fracture, with the increase of Cu content;

(5) The corrosion resistance of the Ti-Cu alloys in SBF solution can be described as: Ti-4Cu alloy > Ti-10Cu alloy > Ti-7Cu alloy. The difference in the electrochemical performance can be attributed to the volume fraction of Ti_2_Cu phases and the “protective barrier” provided by the fine lath structure and uniform distribution of Ti_2_Cu phases.

## Figures and Tables

**Table 1:** Chemical composition of the simulated body fluid (SBF) solution (g/L) [16].

Composition	Concentration	Composition	Concentration
NaCl	8.035	MgCl_2_·6H_2_O	0.311
NaHCO_3_	0.355	CaCl_2_·2H_2_O	0.386
KCl	0.225	Na_2_SO_4_	0.072
K_2_HPO_4_·3H_2_O	0.231		

**Table 2:** EDS analysis results of the precipitated Ti_2_Cu phase marked in Figure 4 (at.%).

Point	Ti	Cu
A	77.32	22.68
B	73.10	26.90
C	82.31	17.69
D	66.59	33.41

**Table 3:** EDS analysis results of the Ti_2_Cu and α-Ti phases in Ti-xCu alloys marked in Figure 6 and Figure 7 (at.%).

Position	Ti	Cu
Area 1	76.15	23.85
Area 2	98.61	1.39
Area 3	67.32	32.68
Area 4	66.33	33.67
Area 5	68.17	31.83
Area 6	98.67	1.33

**Table 4:** Mechanical properties of the as-cast Ti-xCu alloys.

Alloy	YS (MPa)	UTS (MPa)	Strain (%)	HV	Young’s Modulus (GPa)
Ti-4Cu	612.2 ± 1.3	718.5 ± 6.9	6.7 ± 0.4	283 ± 5	103.2
Ti-7Cu	669.1 ± 0.1	801.2 ± 14.4	5.2 ± 1.9	304 ± 16	112.7
Ti-10Cu	749.5 ± 2.2	902.7 ± 25.2	2.6 ± 0.6	329 ± 2	122.7

**Table 5:** Electrochemical parameters of the as-cast Ti-xCu alloys.

Specimen	E_corr_ (V_SCE_)	i_corr_ (μA/cm^2^)
Ti-4Cu	−0.2480	0.043
Ti-7Cu	−0.2243	0.413
Ti-10Cu	−0.1951	0.276

**Table 6:** Electrochemical parameters of the fitting equivalent circuit.

Specimen	R_s_ Ω·cm^2^	R_ct_ MΩ·cm^2^	CPE	
			Y_0_ μF/cm^2^	n
Ti-4Cu	24.5	0.537	48.8	0.708
Ti-7Cu	20.8	0.0528	51.6	0.723
Ti-10Cu	23.4	0.110	36.4	0.765

## Data Availability

The data presented in this study are available on request from the corresponding author.
